# Neighborhood characteristics of expanded access patients at an academic referral center

**DOI:** 10.1017/cts.2025.10115

**Published:** 2025-07-31

**Authors:** Misty Gravelin, Jeanne Wright, Adrian Weyhing, Vikas Kotagal

**Affiliations:** 1 Michigan Investigator Assistance Program, University of Michigan, Ann Arbor, MI, USA; 2 University of Michigan, Precision Health Initiative, Ann Arbor, MI, USA; 3 Department of Neurology, University of Michigan, Ann Arbor, MI, USA; 4 Veterans Affairs (VA) Ann Arbor Healthcare System (VAAAHS), Ann Arbor, MI, USA

**Keywords:** Expanded access, residence characteristics, United states food and drug administration, Michigan, health services accessibility

## Abstract

Expanded access (EA) is a United States regulatory approach for the use of investigational drugs/devices that do not yet have conventional approval, in clinical care contexts. We conducted a retrospective study to analyze the neighborhood characteristics of patients who have received EA treatments at our academic medical center between 2018 and 2023. EA patient neighborhoods showed lower median family income, lower proportion of bachelor’s degree graduates, and a higher proportion of people identifying as non-Hispanic White ethnicity compared to the surrounding (Washtenaw) county. These differences may underly differential interest in EA treatments or may point to disparities in access to evidence-based care.

## Introduction

The US Food and Drug Administration (FDA) created the Expanded Access (EA) pathway to allow the use of investigational drugs, biologics, or devices for clinical treatment [1]. To be eligible to receive a product through EA, a patient must have a serious or life-threatening condition with no satisfactory treatment available, must have the potential for benefit that outweighs the potential risk, and must not have an appropriate clinical trial available [2]. EA fills a critical need for diverse groups of patients: those with rare or recurrent diseases for which treatments or clinical trials may not otherwise exist, patients who have completed clinical trials and require continued therapy, and those who have the misfortune to develop a condition prior to the widespread availability of approved therapies such as in the early days of the COVID-19 pandemic.

However, many barriers exist to treatments with an investigational therapy through EA pathway. The EA treatment pathway is not as widely known as conventional health insurance or patient assistance treatment programs, therefore, a patient has to first find a knowledgeable treating physician to sponsor the request [3,4]. Second, the physician must apply for EA treatment on behalf of the patient and receive the approval from the product manufacturer, the FDA, and a local institutional review board (IRB). These are all very specialized activities that require knowledge and effort on the part of the physician. As a result, EA treatments are often deployed at academic medical centers, where the infrastructure for research and other complex care already exists [5,6].

The University of Michigan (UM) provides institutional support for numerous EA treatments for patients who travel from all areas of the state to receive care. Little is known about the background and socioeconomic characteristics of participants receiving EA treatments. The communities where these patients live may reflect heretofore unreported social determinants of health specific to patients accessing EA treatments. It is possible that EA patients may come from more affluent backgrounds and that access to EA treatments may be a proxy for access to tertiary care. Alternatively, it is possible that patients receiving EA treatments may have health care preferences that differ from other patients with access to tertiary care. We hypothesized that patients receiving EA treatments might come from more affluent environments given the association between higher socioeconomic status and better access to care. Using an existing dataset of neighborhood characteristics linked to our electronic medical record (EMR), we explored the individual and socioeconomic status-linked neighborhood characteristics of patients receiving EA treatments at UM and also compared these characteristics with all the other UM patients living in the surrounding county.

## Methods

We conducted a retrospective cohort study of patients who were enrolled in single-patient, EA treatment plan at UM medical center over a 5-year period between January 1, 2018, and December 31, 2022 (*n* = 124). At UM, all such treatment plans require involvement by the Michigan Investigator Assistance Program (MIAP) which provides support for coordination with clinicians, applications to the United States (US) FDA, local oversight by UM IRB (UM IRBMED), and regulatory compliance. MIAP staff maintain an internal database of EA applications and associated patients at our medical center. A reference group used for comparison of neighborhood data (but not patient-specific demographic data) consisted of all UM patients living in the surrounding county (Washtenaw County, MI) that UM directly serves. This protocol was reviewed by UM IRBMED and designated as meeting Not Regulated status.

First, we collected neighborhood characteristics for both EA and other UM patients from software maintained by the UM Data Office for Clinical and Translational Research (DOCTR) linked to patient identification in the EMR system. Briefly, the US National Neighborhood Data archive (NaNDA) [7] contains data that can be linked to a patient’s recorded place of residence which describes the characteristics of US census tracts. Neighborhood data were available for 110 out of 124 total EA patients (88.7%) and 541,201 UM patients living in Washtenaw county. NaNDA variables are listed in Table 1 and included numerical characteristics of the specific census tract that a given patient resides in. These broad themes of variables describe the relative density of population, ethnic and racial make-up, education, affluence, and other demographic features of the census tract in question. Some NaNDA variables were proportions reflecting a patient’s census tract of record, with values ranging from a minimum score of 0 to a maximum score of 1. In brief, a census tract is a small geographical area within a county and have boundaries designed to be relatively stable over time, allowing for comparisons across censuses. A value of 0 indicates that the specific characteristic is completely absent and a value of 1 indicates that the characteristic is present in 100% of the relevant cases in that particular census tract. It is important to note that these variables do not describe individual patients themselves but rather the neighborhoods they reside in. Second, we collected individual-level information for both EA and other UM patients from DOCTR program. However, only a limited amount of demographic information was available and included age in years, race, and ethnicity, and sex.

**Table 1.** Individual demographic characteristics of expanded access patients and other UM patients

Variable definition	Expanded Access *N* = 124	UM patients *N* = 2,758,783
Age (years)	39.18 (24.39)	47.15 (24.16)
Race*, *n* (%)		
African American	21 (17%)	220,643 (8%)
Asian	5 (4%)	121,702 (4%)
Caucasian	90 (73%)	1,873,976 (68%)
Native Hawaiian/Pacific Islander	1 (<1%)	1,756 (<1%)
Other	7 (6%)	435,501 (16%)
Unknown	5 (4%)	105,205 (4%)
Ethnicity, n(%)		
Hispanic/Latino	3 (2%)	67,714 (2%)
Non-Hispanic/Latino	117 (94%)	1,608,278 (58%)
Unknown/Missing	4 (3%)	1,082,791 (39%)
Sex, n (%)		
Female	58 (47%)	1,474,450 (53%)
Male	66 (53%)	1,282,904 (47%)
Unknown/Missing	0	1,429 (<1%)

* *Note:* Patients were able to identify as more than one racial group.

Descriptive statistics including means, counts, and standard deviations were used to characterize the distribution of variables in the two groups. The characteristics between EA patients and all other UM patients from Washtenaw County were compared using unpaired two-sample t-tests. Given the exploratory nature of this study, a *p*-value of <0.05 was used to define intergroup differences. Mean age between groups was compared in a Washtenaw County sample with rare ages >100 removed given the potential for coding errors in the underlying values and with age at the date of April 24, 2024, used as an anchor date. Given the potential for type-1 error, we would encourage any intergroup differences to be interpreted as hypothesis generating in nature and not definitive.

## Results

Table 1 demonstrates the individual-level demographic information of EA and other patients served by UM medical center, as well as inter-group comparisons. EA patients in general were younger than Washtenaw County patients but did not differ significantly in race, ethnicity, or sex.

The neighborhood characteristics of 110 EA patients and the reference population of UM patients residing in the Washtenaw county for whom NaNDA data was available, are demonstrated in Table 2. EA patient neighborhoods showed some patterns of differences from Washtenaw County patient neighborhoods. EA patient neighborhoods had slightly lower neighborhood affluence with a median neighborhood income about 20% lower than Washtenaw County patients. EA patient neighborhoods showed a lower density of persons per square mile, which we interpreted as a proxy for neighborhood rurality. Some of these intergroup differences were driven by different median wealth values among non-Hispanic white neighborhood residents. EA patient neighborhoods tended to have a lower proportion of younger adults and higher proportion of older adults. Relatively speaking, EA neighborhoods tended to have a lower proportion of aggregate “Hispanic, foreign-born, or non-English speakers.” EA neighborhoods also tended to have fewer college educated adults.

**Table 2.** Neighborhood characteristics of expanded access patients and other UM patients from Washtenaw county

Variable definition	Expanded Access *N* = 110*	Washtenaw County *N* = 541,201*	*p*-value
Census land area, miles^2^	11.54 (18.46)	6.64 (14.27)	*p* < 0.01
Total population of the census tract	3,862.75 (1461.34)	4,071.35 (1,491.02)	*p* = 0.14
Persons/mile^2^	2,141.97 (2,365.87)	3,936.31 (4,492.17)	*p* < 0.01
***Demographics, Race, and Ethnicity, Mean Percent (Standard deviation)***			
Age 15+ Never Married	31.33% (.10)	42.03% (.21)	*p* < 0.01
Under 18 years	22.05% (.06)	18.36% (.08)	*p* < 0.01
18-29 years	14.96% (.06)	26.96% (.23)	*p* < 0.01
30-39 years	11.60% (.03)	13.00% (.05)	*p* < 0.01
40-49 years	12.59% (.04)	10.81% (.05)	*p* < 0.01
50-69 years	27.30% (.06)	21.64% (.09)	*p* < 0.01
70+ years	11.50% (.06)	9.24% (.06)	*p* < 0.01
Hispanics	4.61% (.06)	4.82% (.03)	*p* = 0.53
Non-Hispanic White	77.64% (.23)	68.01% (.18)	*p* < 0.01
Non-Hispanic Black	10.38% (.19)	11.19% (.14)	*p* = 0.56
Foreign born	6.86% (.09)	14.82% (.12)	*p* < 0.01
Age 5+ who do not speak English well	1.12% (.02)	1.70% (.02)	p = 0.01
Hispanic, Foreign Born, and/or limited English	4.20% (.04)	7.11% (.05)	*p* < 0.01
***Education, Income, and Age***			
Less than High School Diploma	7.42% (.06)	4.07% (.04)	*p* < 0.01
High School Diploma and/or Some College	58.62% (.15)	34.89% (.19)	*p* < 0.01
Bachelor’s Degree or Higher	33.96% (.19)	61.03% (.22)	*p* < 0.01
Families with income less than $40,000	18.94% (.13)	17.34% (.15)	*p* = 0.27
Families with income $40,000 to $74,999	25.37% (.11)	20.27% (.11)	*p* < 0.01
Families with income $75,000 to $124,999	28.37% (.09)	24.60% (.11)	*p* < 0.01
Families with income $125,000 or more	27.32% (.18)	37.79% (.21)	*p* < 0.01
16+ civilian labor force unemployed	5.52% (.04)	4.36% (.04)	*p* < 0.01
Income below poverty level in the past 12 months	10.06% (.09)	16.17% (.18)	*p* < 0.01
Households with public assistance income or food stamps	10.99% (.11)	7.43% (.08)	*p* < 0.01
Owner Occupied Housing Units	76.21% (.20)	58.94% (.31)	*p* < 0.01
Female-headed families w/kids	8.77% (.08)	7.48% (.09)	*p* = 0.12
Single parent families w/kids	11.89% (.09)	10.06% (.10)	*p* = 0.05
Family Income	$88,610.6 (SD: 32,207.88) *n* = 108	$109,654.4 (SD: 44,277.95)	*p* < 0.01

*The available n for denominator for Washtenaw county patients were 541,072 for race and ethnicity, age, and education distribution; 540,685 for socioeconomic status; 538,167 for family income and type description; 507,632 for overall family income. The available n for denominator for expanded access patients was 110 for most of the variables except 108 for overall family income.

**SD = Standard Deviation.

## Discussion

EA patients at the UM are younger and come from neighborhoods that are less affluent, less diverse, and more rural than UM patients in the suburban county surrounding UM. These findings stand in contrast to our underlying hypothesis that access to EA treatments would be associated with greater affluence. It is possible that these trends reflect medical center-specific factors including regional variation in the availability and coverage specifics of health insurance, access to competing health care systems for primary and specialty care, regional idiosyncrasies related to patient preferences, and/or normative practices among medical providers at our center. Alternatively, findings from our study – drawn from several years of internally tracked data on EA applications to the US FDA – may shed light on a specific health disparity unique to patients receiving care at academic referral centers in the United States: that rural patients coming from racially homogenous regions may have an easier time gaining access to EA treatments than urban patients coming from more diverse communities. On the one hand, EA access might be a marker for greater provider engagement in patient care [8], given it prioritizes access to rarely used therapeutic approaches. On the other hand, putative treatments given in an EA access are less likely to be evidence-based [9] and may confer an unfavorable risk-benefit ratio. Determining whether these findings are replicable at other medical centers or whether they differ depending on regional context, is an important next step.

The trends seen in the neighborhood data that we present herein do not describe the patients themselves but rather the communities in which they live. For example, we report that EA patients were younger on average than patients living in Washtenaw country while also reporting that the proportion of young adults in the neighborhoods of EA patients was lower than that of Washtenaw County. In this specific example, this difference is likely driven by the fact that many of the patients receiving EA treatments at our medical center are pediatric. Nevertheless, the difference between patient-specific factors (which would reveal clinical and demographic characteristics of the cohort itself) and neighborhood factors (which describe the socioeconomic environment of the community that the patient comes from) has implications on how these data should be interpreted.

The published literature is mixed in its descriptions of the demographic factors that characterize patients receiving EA treatments. Darrow and colleagues have raised concerns regarding equity of access to EA treatments, arguing that only the most affluent patients are likely to receive EA treatments [3]. The commonly discussed cases of EA, including Josh Hardy [10] and Abigail Burrows [11], support this assumption, but these anecdotes may not necessarily align with the experiences of other EA patients. Concern about equity in the access to EA treatments is heightened by the reality that the FDA’s Right to Try does not require drug manufactures to cover the cost of the treatment itself (although many manufacturers still do). Such treatments are not typically covered by medical insurance and can lead to substantial out of pocket costs for patients and families [12]. Alternatively, EA patients are generally expected to have a serious or life-threatening illness, which may deleteriously impact their household wealth and earning potential.

The census-tract-level outcomes measured in this project allow comparison of EA patient neighborhoods to those of a comparator group. This means we can draw stronger inferences about the communities that EA patients reside in than we can about the demographic characteristics of EA patients themselves. Nevertheless, the intergroup differences in neighborhood factors found in our dataset are in line with an emerging area of public health research identifying that neighborhood factors influence key health mediators in acute [13] and chronic disorders [14] throughout the US. Such neighborhood-focused associative factors touch many broad categories including air and environmental quality, neighborhood poverty and disinvestment affecting green spaces, sidewalks and parks, and the paucity of local grocery stores or pharmacies [15].

Easy access to EA treatments might be a marker for good healthcare or a marker for substandard healthcare. Use of EA treatment inherently involves a deviation from established evidence-based medical practice. One can easily imagine a situation where such a deviation might be undertaken at the discretion of either the patient/family or the medical provider. It is likely that the severity of the disease in question along with underlying attitudes towards risk aversion and heuristic decision making [16] influence differential interest in pursuing EA treatments. For example, a survey of US oncologists who have experience using the EA treatment pathway shows that such providers generally put more weight on the balance of clinical risks and benefits of a treatment compared to the potential burden such a treatment might represent for a patient/family [8]. Interestingly, the same study showed that these providers had a high degree of confidence in their ability to weigh these risks and benefits, despite the relative lack of evidence-based data for off label EA treatments. A similar survey study conducted in the Netherlands of patients with life-threatening illness revealed that, even though most patients surveyed had limited familiarity with such investigational nonstandard treatments, they nevertheless felt strongly that clinical decisions about whether to pursue such treatments were highly personal in nature. Survey respondents also articulated a high willingness to pay out of pocket for such nonstandard treatments [17]. Collectively, these studies paint a picture of a potentially vulnerable set of patients and providers that span many different disease conditions.

Our study has several key limitations that should be considered when interpreting our findings. First, as mentioned previously, we did not analyze intergroups differences in patient-specific demographic factors. Second, a small subset of EA patients did not have NaNDA data available (11.3%) for analysis. The same is true of the non-EA patients residing in Washtenaw county shown in Table 2. It is subsequently possible that information bias may be influencing our findings. Third, we did not obtain detailed clinical information on EA patients that might allow us to conduct subgroup analyses by condition. Finally, our enrollment window spans a time period (2018–2022) where most medical practices underwent substantial logistical change in the context of the COVID-19 pandemic. Nevertheless, our study also has several key strengths. The breadth and rigor of case ascertainment used to identify EA patients in real-time over a 5-year period was made possible by careful record keeping by members of our NIH-supported CTSA. Second, the use of neighborhood data derived from a comparator arm within the county that UM serves is unique and allows us to ask novel questions about how EA patients might differ from representative comparator groups also receiving care at our institution.

Factors that influence the use and deployment of EA treatments reflect unique facets of the American health care system and have the potential to exacerbate health disparities through inequitable access and application. Studying factors associated with EA treatments in other academic and community-based settings throughout the USA would be important next steps in determining whether greater regulatory scrutiny of this process is indicated.

## References

[1] US Food and Drug Administration. Individual patient expanded access applications: form FDA 3926. Guidance for Industry. 2017, (https://www.fda.gov/downloads/Drugs/GuidanceComplianceRegulatoryInformation/Guidances/UCM432717.pdf) Accessed August 6, 2025.

[2] Food and Drug Administration DoHaHS. 21 CFR 312.305, (https://www.ecfr.gov/current/title-21/part-312/section-312.300) Accessed January 9, 2025

[3] Darrow JJ , Sarpatwari A , Avorn J , et al. Practical, legal, and ethical issues in expanded access to investigational drugs. N Engl J Med. 2015;372:279–286. doi: 10.1056/NEJMhle1409465. 25587952

[4] Zettler ME , Jeune-Smith Y , Feinberg BA , et al. Expanded access and right to try requests: the community oncologists experience. JCO Oncol Pract. 2021;17:e1719–e1727. doi: 10.1200/op.20.00569. 33886355 PMC8600511

[5] Lee I , Simmons Z. Hopes and concerns regarding the implementation of expanded access protocols in amyotrophic lateral sclerosis. Muscle Nerve. 2023;67:433–435. doi: 10.1002/mus.27828. 36999228

[6] Yerton M , Winter A , Gelevski D , et al. Expanded access protocol (EAP) program for access to investigational products for amyotrophic lateral sclerosis (ALS). Muscle Nerve. 2023;67:456–463. doi: 10.1002/mus.27819. 36929648

[7] Melendez R , Clarke P , Khan A , Gomez-Lopez I , Li M , Chenoweth M . National Neighborhood Data Archive (NaNDA): Socioeconomic Status and Demographic Characteristics of Census Tracts, United States, 2008-2017. Ann Arbor, MI: Inter-university Consortium for Political and Social Research [distributor]; 2020.

[8] Gould P , Salam T , Kimberly L , et al. Perspectives of academic oncologists about offering expanded access to investigational drugs. JAMA Netw Open. 2022;5:e2239766. doi: 10.1001/jamanetworkopen.2022.39766. 36318206 PMC9627412

[9] Jarow JP , Lurie P , Ikenberry SC , et al. Overview of FDAs expanded access program for investigational drugs. Ther Innov Regul Sci. 2017;51:177–179. doi: 10.1177/2168479017694850. 28553565 PMC5443564

[10] Larcker DF , Larcker S , Tayan B. Josh Hardy and the# saveJosh army: How corporate risk escalates and accelerates through social media. *Rock Center for Corporate Governance at Stanford University Closer Look Series: Topics, Issues and Controversies in Corporate Governance and Leadership No. CGRP-40*, 2014.

[11] Fountzilas E , Said R , Tsimberidou AM. Expanded access to investigational drugs: balancing patient safety with potential therapeutic benefits. Expert Opin Investig Drugs. 2018;27:155–162. doi: 10.1080/13543784.2018.1430137. PMC6291242 29353505

[12] Carrieri D , Peccatori FA , Boniolo G. The ethical plausibility of the Right to try’ laws. Crit Rev Oncol Hematol. 2018;122:64–71. doi: 10.1016/j.critrevonc.2017.12.014. 29458791

[13] Hyun KK , Brieger D , Woodward M , et al. The effect of socioeconomic disadvantage on prescription of guideline-recommended medications for patients with acute coronary syndrome: systematic review and meta-analysis. Int J Equity Health. 2017;16:162. doi: 10.1186/s12939-017-0658-z. 28859658 PMC5579970

[14] Fuemmeler BF , Shen J , Zhao H , Winn R. Neighborhood deprivation, racial segregation and associations with cancer risk and outcomes across the cancer-control continuum. Mol Psychiatry. 2023;28:1494–1501. doi: 10.1038/s41380-023-02006-1. 36869227

[15] Musaogullari A , Moorhead J , Plana A , Johnson A. Space for improvement: ZIP codes should not determine cardiovascular longevity, a scoping review. Trends Cardiovasc Med. 2025;35:214–218. doi:10.1016/j.tcm.2024.12.005. 39667536 PMC12052493

[16] Nierenberg AA , Smoller JW , Eidelman P , et al. Critical thinking about adverse drug effects: lessons from the psychology of risk and medical decision-making for clinical psychopharmacology. Psychother Psychosom. 2008;77:201–208. doi: 10.1159/000126071. 18418026

[17] Bunnik EM , Aarts N. What do patients with unmet medical needs want? A qualitative study of patients views and experiences with expanded access to unapproved, investigational treatments in the Netherlands. BMC Med Ethics. 2019;20:80. doi: 10.1186/s12910-019-0420-8. 31706313 PMC6842468

